# Interaction between miR-142-3p and *BDNF* Val/Met Polymorphism Regulates Multiple Sclerosis Severity

**DOI:** 10.3390/ijms25105253

**Published:** 2024-05-11

**Authors:** Ettore Dolcetti, Alessandra Musella, Sara Balletta, Luana Gilio, Antonio Bruno, Mario Stampanoni Bassi, Gianluca Lauritano, Fabio Buttari, Diego Fresegna, Alice Tartacca, Fabrizio Mariani, Federica Palmerio, Valentina Rovella, Rosangela Ferese, Stefano Gambardella, Emiliano Giardina, Annamaria Finardi, Roberto Furlan, Georgia Mandolesi, Diego Centonze, Francesca De Vito

**Affiliations:** 1 Neurology Unit, IRCCS Neuromed, 86077 Pozzilli, Italy; ettoredolcetti@hotmail.it (E.D.); balletta.sara@gmail.com (S.B.); gilio.luana@gmail.com (L.G.); antonio.bruno91@yahoo.it (A.B.); mario_sb@hotmail.it (M.S.B.); gianlucalaurita@gmail.com (G.L.); fabio.buttari@gmail.com (F.B.); ferese.rosangela@gmail.com (R.F.); stefanogambardella@gmail.com (S.G.); 2 Ph.D. Program in Neuroscience, Department of Systems Medicine, University of Rome Tor Vergata, 00133 Rome, Italy; alicetarti@gmail.com (A.T.); federicapalmerio@gmail.com (F.P.); 3 Synaptic Immunopathology Laboratory, IRCCS San Raffaele Roma, 00163 Rome, Italy; alessandra.musella@uniroma5.it (A.M.); diego.fresegna@sanraffaele.it (D.F.); georgia.mandolesi@uniroma5.it (G.M.); 4 Department of Human Sciences and Quality of Life Promotion, University of Rome San Raffaele, 00163 Rome, Italy; 5 Faculty of Psychology, Uninettuno Telematic International University, 00186 Rome, Italy; 6 Department of Systems Medicine, Tor Vergata University, 00133 Rome, Italy; marianifabrizio02@gmail.com (F.M.); valentina.rovella@uniroma2.it (V.R.); 7 Department of Biomolecular Sciences, University of Urbino “Carlo Bo”, 61029 Urbino, Italy; 8 Genomic Medicine Laboratory, IRCCS Fondazione Santa Lucia, 00179 Rome, Italy; emiliano.giardina@uniroma2.it; 9 Department of Biomedicine and Prevention, University of Rome “Tor Vergata”, 00133 Rome, Italy; 10 Clinical Neuroimmunology Unit, Institute of Experimental Neurology (INSpe), Division of Neuroscience, IRCCS San Raffaele Scientific Institute, 20132 Milan, Italy; finardi.annamaria@hsr.it (A.F.); furlan.roberto@hsr.it (R.F.); 11 Faculty of Medicine and Surgery, Vita e Salute San Raffaele University, 20132 Milan, Italy

**Keywords:** microRNA, synaptopathy, neuroinflammation, synapsis, proinflammatory cytokine, biomarker

## Abstract

MiR-142-3p has recently emerged as key factor in tailoring personalized treatments for multiple sclerosis (MS), a chronic autoimmune demyelinating disease of the central nervous system (CNS) with heterogeneous pathophysiology and an unpredictable course. With its involvement in a detrimental regulatory axis with interleukin-1beta (IL1β), miR-142-3p orchestrates excitotoxic synaptic alterations that significantly impact both MS progression and therapeutic outcomes. In this study, we investigated for the first time the influence of individual genetic variability on the miR-142-3p excitotoxic effect in MS. We specifically focused on the single-nucleotide polymorphism Val66Met (rs6265) of the brain-derived neurotrophic factor (*BDNF*) gene, known for its crucial role in CNS functioning. We assessed the levels of miR-142-3p and IL1β in cerebrospinal fluid (CSF) obtained from a cohort of 114 patients with MS upon diagnosis. By stratifying patients according to their genetic background, statistical correlations with clinical parameters were performed. Notably, in Met-carrier patients, we observed a decoupling of miR-142-3p levels from IL1β levels in the CSF, as well as from of disease severity (Expanded Disability Status Score, EDSS; Multiple Sclerosis Severity Score, MSSS; Age-Related Multiple Sclerosis Severity Score, ARMSS) and progression (Progression Index, PI). Our discovery of the interference between *BDNF* Val66Met polymorphism and the synaptotoxic IL1β-miR-142-3p axis, therefore hampering miR-142-3p action on MS course, provides valuable insights for further development of personalized medicine in the field.

## 1. Introduction

MS represents one of the most common autoimmune disabling diseases of the CNS, characterized by synaptic damage, demyelination, and early neuronal death mediated by neuroinflammation [1,2]. MS manifests in various clinical forms, with the relapsing-remitting MS (RRMS) being the most common [1]. The pathophysiology of RRMS involves varying degrees of acute and chronic inflammation and neurodegeneration, and the intricate interplay of genetic and epigenetic factors contributing to the heterogeneity of the disease [3]. Patients with RRMS (pwRRMS) experience alternating periods of clinical manifestations and remission, eventually transitioning into the progressive form marked by irreversible and advancing disability [1]. Many investigations have indicated that excitotoxic synaptic damage can contribute to the silent MS progression [2,4].

The molecular triggers of MS synaptic alterations include not only proinflammatory cytokines but also microRNAs (miRNA). Among these post-transcriptional regulators of gene expression, miR-142-3p stands up for importance in MS excitotoxicity [5,6,7]. The dual nature of miR-142-3p has clearly emerged due to its action on both immune and synaptic compartments. Its role in modulating immune responses and neuroinflammation has been well established for many years [8,9]. More recently, MS preclinical and clinical studies have revealed that miR-142-3p is part of a detrimental regulatory axis with the pro-inflammatory cytokine IL-1β, which contributes to excitotoxic synaptic overstimulation with serious consequences for disease prognosis and the efficacy of disease-modifying treatments [5,6]. Compelling evidence from a chimeric ex vivo MS model showed that high levels of miR-142-3p in the human CSF can impair synaptic transmission in healthy murine brain slices, mimicking synaptic damage typically observed in experimental MS [5].

However, the complete understanding of the molecular interactors of miR-142-3p in the MS excitotoxic pathway and the influence of individual genetic variability on miR-142-3p action remains elusive. Notably, miR-142-3p has been recently demonstrated to indirectly affect the transcription of *Bdnf* by targeting the calmodulin-kinase 2 (*Camk2a*) mRNA in in vitro activated microglia [10].

BDNF is a pivotal neurotrophin that has garnered substantial attention in MS also due to its potential implications in inflammatory synaptic dysfunctions [11].

The *BDNF* gene, located on chromosome 11 in humans [12], produces many transcripts by combining nine exons, each with its own promoter triggered by different stimuli [13]. Each mRNA isoform consists of two exons and encodes for the same BDNF protein, but their abundance varies across different brain regions. Additionally, the primary *BDNF* transcripts can be processed at two alternative polyadenylation sites, resulting in mature mRNAs with short or long 3′UTRs of different lengths [14,15]. Neuronal activity heavily influences most of the regulatory mechanisms controlling BDNF expression [16]. Furthermore, BDNF is initially synthesized as the proBDNF precursor protein, which is cleaved by intracellular or extracellular peptidase enzymes into mature BDNF and propeptide [17].

BDNF secretion at the synapsis is altered in neuroinflammation [18] and its action is dependent from single-nucleotide polymorphisms (SNPs), such as *BDNF* Val66Met (rs6265). This SNP is in the last exon, present in all BDNF transcripts, and represents the most extensively studied SNP in the BDNF gene. The substitution of a valine (Val) to methionine (Met) at position 66 of the proBDNF protein has been associated with numerous impaired functions of the CNS, moving from neuronal survival and synaptic plasticity to the modulation of immunophenotypes in resident and non-resident cells [19,20,21,22,23].

Our study aims to evaluate the clinical significance of the interaction between CSF miR-142-3p levels and the Val66Met polymorphism in pwRRMS at diagnosis, considering its potential prognostic significance.

## 2. Results

### 2.1. CSF miR-142-3p Associates with Adverse Clinical Signs of RRMS

In order to assess the clinical relevance of miR-142-3p in the early phases of MS, we evaluated its CSF levels at diagnosis in a cohort of 114 pwRRMS (Table 1) and explored its associations with clinical and demographical characteristics.

**Figure 1:**
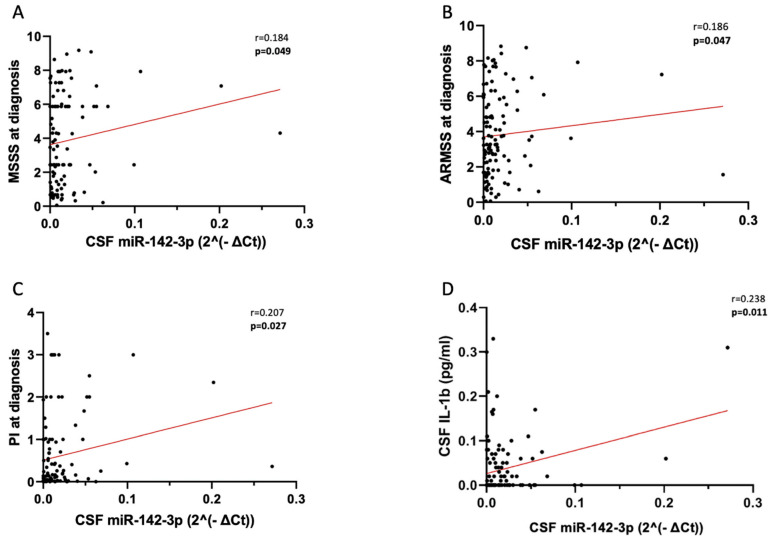
Correlation analysis between CSF levels of miR-142-3p and clinical parameters in pwRRMS. Correlation plots show positive correlation between CSF levels of miR-142-3p and indexes of disease severity (**A**,**B**), disease progression (**C**) and neuroinflammatory excitotoxicity (**D**). MiR-142-3p values were normalized to miR-204-5p using the ΔCt calculation (‘Ct_miR-142-3p_’ − ‘Ct_miR-204-5p_’). Bold denotes a statistical significance, *p* < 0.05. Legend: CSF (cerebrospinal fluid); MSSS (Multiple Sclerosis Severity Scale); ARMSS (Age-Related Multiple Sclerosis Severity Score); PI (progression index); IL-1β (interleukin-1beta).

We observed a significant positive correlation between CSF miR-142-3p levels, and key indicators of disease severity, like MSSS (Figure 1A: Spearman’s Rho = 0.184, *p* = 0.049) and ARMSS (Figure 1B: Spearman’s Rho = 0.186, *p* = 0.047). The levels of miR-142-3p in the CSF were also correlated with disease progression, as estimated by PI (Figure 1C: Spearman’s Rho 0.207, *p* = 0.027). No significant associations with other clinical and demographical parameters were found (age at diagnosis: *p* = 0.335, sex *p* = 0.063, disease duration: *p* = 0.441, EDSS *p* = 0.061, radiological activity *p* = 0.371, presence of OCB *p* = 0.807).

Moreover, we confirm the presence of the detrimental regulatory axis IL-1β-miR-142-3p in the CSF of pwRRMS, as evidenced by a direct correlation between the levels of these two excitotoxic molecules in the biofluid (Figure 1D: Spearman’s Rho = 0.238, *p* = 0.011), consistent with previous findings established in a more heterogenous cohort of patients [6].

### 2.2. Met66-Allele Is Not Associated with the Risk of RRMS Onset but Can Contribute to Disease Course Heterogeneity

**Figure 2:**
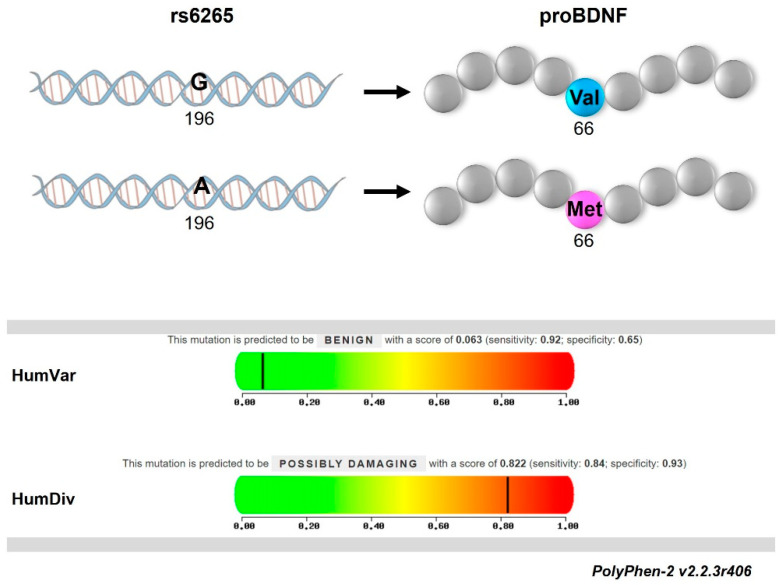
Bioinformatic prediction of Val66Met SNP pathogenicity by PolyPhen-2 v2.2.3r406. HumVar prediction revealed no pathogenic effects of the missense mutation on Mendelian diseases, while HumDiv predicted a possible influence in non-Mendelian diseases. No effect (benign), score = 0.00–0.15; Possible effect (damaging), score = 0.16–1.00. Legend: rs6256 (G > A genetic variant; Val66Met missense substitution); proBDNF (pro-protein encoded by Brain-Derived Neurotrophic Factor gene).

The genetic screening of pwRRMS participating in our study for the SNP *BDNF* Val66Met revealed an aligned distribution of Val-allele (frequency of G = 78.51%) and Met-allele (frequency of A = 21.49%) with general Caucasian population (chi-square n.s. *p* = 0.499). Genotype frequencies were in Hardy–Weinberg equilibrium (chi-square n.s. *p* = 0.695) with 60.52% Val66 homozygotes (GG, *n* = 69), 35.96% heterozygotes (AG, *n* = 41) and 3.50% Met66 homozygotes (AA, *n* = 4). Coherently, the pathogenicity prediction of Val66Met substitution by PolyPhen-2 algorithm (Figure 2) indicated no drastic effects of the SNP on the risk of diseases from all the remaining genetic variations (HumVar score = 0.06; sensitivity = 0.92; specificity = 0.65) but suggested its involvement in the endophenotypes of multifactorial diseases such as MS (HumDiv score = 0.82; sensitivity = 0.84; specificity = 0.93).

### 2.3. The IL1β-miR-142-3p Axis Is Disrupted in pwRRMS Carrying Met-Allele

The well-established synaptic effect of Val66Met substitution in proBDNF [19,20] and the recently discovered link between *BDNF* and miR-142-3p in neuroinflammation [10] prompted us to investigate the SNP influence on the excitotoxic IL1β-miR-142-3p axis in the CSF of pwRRMS. To this aim, we examined separately the Val66 homozygotes (Val/Val; Table 2) and the Met-carriers (Val/Met and Met/Met; Table 3) present in our cohort. These two groups of patients were equivalent across all demographic and clinical characteristics assessed at diagnosis, with exception of age (Val/Val, median [IQR] = 31.3 [25.1–41.7], Met-allele carriers, median [IQR]: = 39.8 [31.5–47.2], Mann–Whitney test *p* = 0.010). CSF values of miR-142-3p and IL-1β were unchanged in the two conditions (CSF miR-142-3p: Val/Val median [IQR] = 0.009 [0.004–0.020], Met-allele median [IQR] = 0.009 [0.003–0.020], Mann–Whitney test *p* = 0.683; CSF IL-1β: Val/Val median [IQR] = 0.0002 [0.000–0.060], Met-allele median [IQR] = 0.0001 [0.000–0.030], Mann–Whitney test *p* = 0.209).

**Figure 3:**
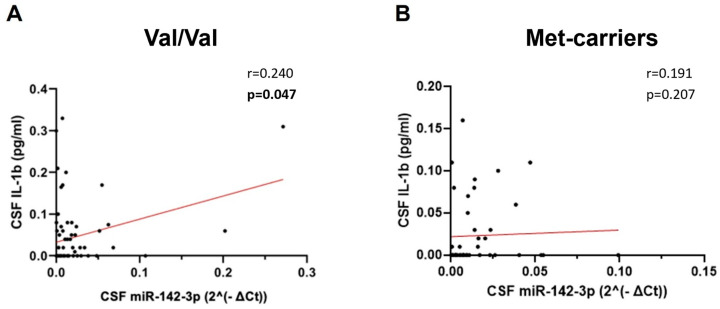
Correlation analysis between CSF levels of miR-142-3p and IL-1β according to *BDNF* Val66Met variants. Correlation plots show a positive association between the levels of CSF miR-142-3p and IL-1β in Val/Val group (**A**) but not in Met-carrier group (**B**). miR-142-3p values were normalized to miR-204-5p using the ΔCt calculation (‘Ct_miR-142-3p_’ − ‘Ct_miR-204-5p_’). Bold denotes a statistical significance *p* < 0.05. Legend: CSF (cerebrospinal fluid); Val/Val (Val66 homozygotes); Met-carriers (Met66 homozygotes and heterozygotes); IL-1β (interleukin-1beta).

In Val/Val patients (Table 2), we observed a significant direct correlation between the CSF levels of miR-142-3p and IL-1β (Figure 3A; Spearman’s Rho = 0.240, *p* = 0.047, *n* = 69), as there was in the whole cohort of patients (Figure 1D). Conversely, the association between these molecules was lost in the Met-carrier group (Table 3; Figure 3B; Spearman’s Rho = 0.191, *p* = 0.207, *n* = 69 45), indicating the disruption of the regulatory axis IL1β-miR-142-3p.

### 2.4. The Detrimental Effect of miR-142-3p on Clinical Parameters Is Impaired in pwRRMS Carrying Met-Allele

We evaluated the miR-142-3p-associated clinical manifestations in pwRRMS, stratified based on *BDNF* Val66Met variants.

**Figure 4:**
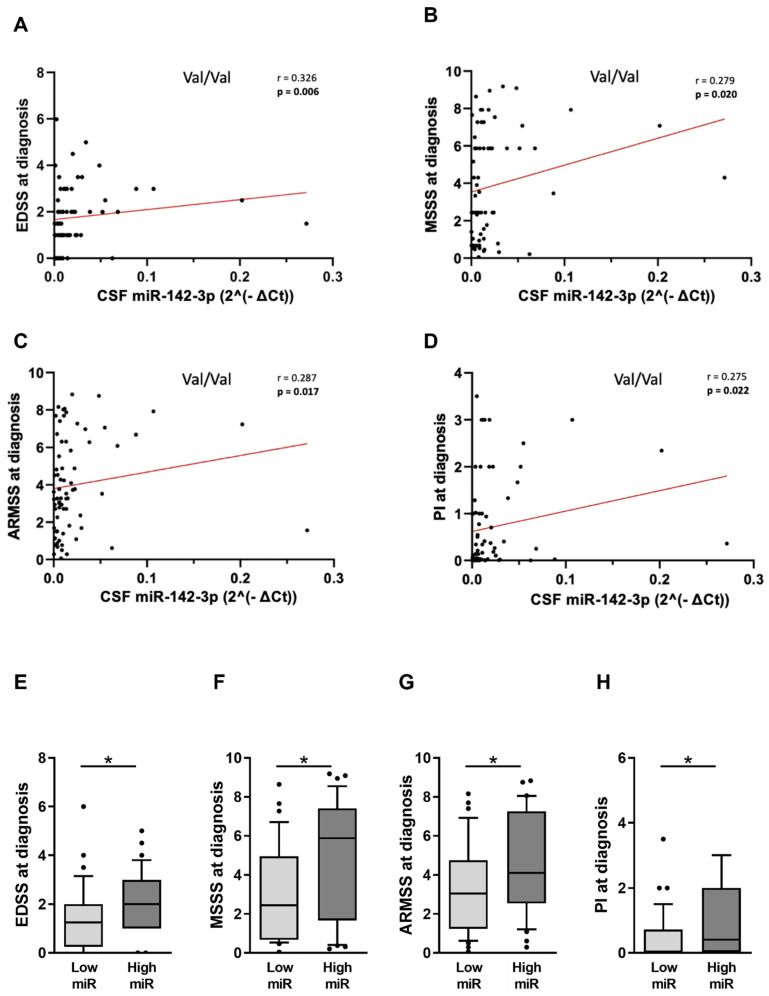
Statistical analysis of the interaction between CSF miR-142-3p levels and RRMS clinical indexes in Val/Val group. (**A**–**D**) Correlation plots show positive correlation between progression and severity parameters of disease and CSF miR-142-3p in Val/Val group. (**E**–**H**) Box plots show that high CSF levels of miR-142-3p are associated with the more severe values of EDSS, MSSS, ARMSS and PI in the Val/Val group. Legend: CSF (cerebrospinal fluid); EDSS (Expanded Disability Status Scale); MSSS (Multiple Sclerosis Severity Scale); ARMSS (Age-Related Multiple Sclerosis Severity Score); PI (progression index); CSF miR-142-3p values were normalized to miR-204-5p using the ΔCt calculation (‘Ct_miR-142-3p_’ − ‘Ct_miR-204-5p_’); low miR (<0.010), high miR (≥0.010); * denotes *p* < 0.05.

In Val/Val condition (Table 2), CSF miR-142-3p levels at diagnosis exhibited direct correlation with EDSS (Figure 4A: Spearman’s Rho = 0.326, *p* = 0.006; *n* = 69 69), MSSS (Figure 4B: Spearman’s Rho 0.279, *p* = 0.020, *n* = 69 69), ARMSS (Figure 4C: Spearman’s Rho 0.287, *p* = 0.017, *n* = 69 69) and PI (Figure 4D: Spearman’s Rho = 0.275, *p* = 0.022; *n* = 69 69), consisted with the preserved IL1β-miR-142-3p axis (Figure 3A).

Upon dividing patients according to CSF miR-142-3p levels (cut-off: Low miR < 0.01, *n* = 69 36; High miR ≥ 0.01, *n* = 69 33), we observed that patients with higher miRNA, showed significantly higher indexes of both disease severity (Figure 4E–G; EDSS: high miR group, median [IQR]: = 2 [1–3] vs. low miR group, median [IQR] = 1.5 [0–2], *p* = 0.013; MSSS: high miR group, median [IQR]: = 5.9 [1.7–7.4] vs. low miR group, median [IQR] = 2.4 [0.7–4.9], *p* = 0.022; ARMSS: high miR group, median [IQR]: = 4.1 [2.5–7.3] vs. low miR group, median [IQR] = 3 [1.2–4.7], *p* = 0.026) and disease progression (Figure 4H; PI high miR group, median [IQR]: = 0.4 [0.0.2–2] vs. low miR group, median [IQR] = 0.04 [0–0.7], *p* = 0.011) compared to the patients with lower miR-142-3p levels.

**Figure 5:**
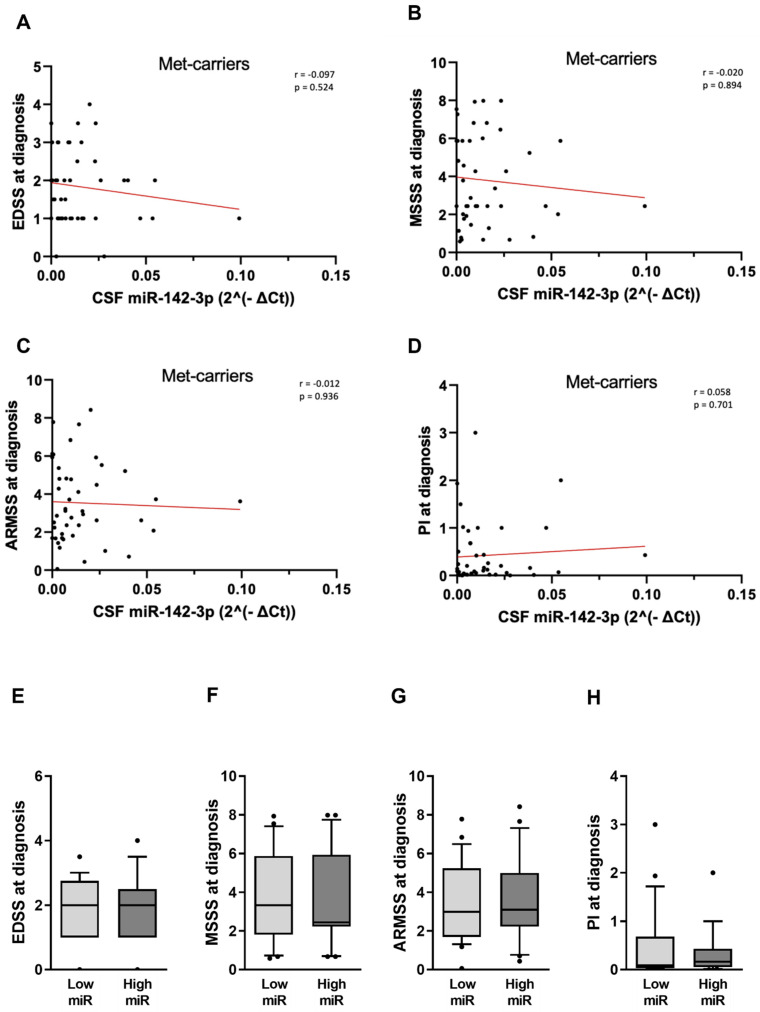
Statistical analysis of the interaction between CSF miR-142-3p levels and RRMS clinical indexes in Met-carrier group. (**A**–**D**) Correlation plots show no correlation between the progression parameters of disease and CSF miR-142-3p in Met-carrier group. (**E**–**H**) Box plots show no difference in EDSS, MSSS, ARMSS, and PI values in Met-carrier group according to CSF levels of miR-142-3p. Legend: CSF (cerebrospinal fluid); EDSS (Expanded Disability Status Scale); MSSS (Multiple Sclerosis Severity Scale); ARMSS (Age-Related Multiple Sclerosis Severity Score); PI (progression index); CSF miR-142-3p values were normalized to miR-204-5p using the ΔCt calculation (‘Ct_miR-142-3p_’ − ‘Ct_miR-204-5p_’); low miR (<0.010), high miR (≥0.010); no statistical significance *p* > 0.05.

Importantly, miR-142-3p was not correlated with any disease parameters in Met-carriers (Table 3; Figure 5A–D: EDSS: *p* = 0.524; MSSS: *p* = 0.894; ARMSS: *p* = 0.936; PI: *p* = 0.701). No differences emerged even upon patients’ stratification according to miR-142-3p levels (Figure 5E–H; EDSS, *p* = 0.716; PI, *p* = 0.641; MSSS, *p* = 0.973; ARMSS, *p* = 0.794).

## 3. Discussion

This study explored the role of CSF miR-142-3p as a biomarker for MS course, particularly in relation to the *BDNF* Val66Met polymorphism. Our focus was on the early phases of the disease, analyzing pwRRMS at the time diagnosis because the identification of valuable prognostic factors at this stage is urgent for guiding therapeutic choices in the expanding treatment repertoire.

We previously demonstrated in a heterogenous cohort of patients with MS that miR-142-3p is a key regulator of synaptopathy-driven disease progression, which acts as molecular effector of excitotoxic synaptic alterations induced by IL-1β [5,6].

Here, we discovered that the CSF miR-142-3p levels were directly correlated with the different aspects of the RRMS course by evaluating various clinical parameters. EDSS scoring [24] allowed us to assess the disability status of patients. To account for age-related factors, EDSS corrections using ARMSS was applied, as proposed by Alrouji and colleagues [25]. Additionally, MSSS provided indications on the disease disability normalized to disease duration [26]. The rate of disability progression was further evaluated using PI [27].

The assessment of the interplay between genetic variants and an excitotoxic miRNA in determining the clinical manifestations of RRMS represents the primary novelty of this work. Interestingly, we showed that the severity and progression of multiple sclerosis (MS) were not influenced by the CSF levels of miR-142-3p in patients with *BDNF* Met66-allele background, whereas Val/Val patients with high levels of miR-142-3p exhibited a more severe phenotype.

*BDNF* Val66Met polymorphism has been described to exert a dichotomous role in MS. Some studies showed a protective role of this polymorphism on the risk of progression in the later stages of the disease [28,29]. Other evidence sustained a detrimental role in early MS stages, with higher radiological activity, lower gray-matter volumes, and higher concentrations of a specific subset of CSF proinflammatory cytokines [21,22]. In our study, we observed that Met-allele, but not the Val/Val genotype, uncoupled the levels of miR-142-3p and IL-1β in the CSF of pwRRMS even though the values were not influenced by the genetic background. This was consistent with the loss of miR-142-3p-dependent disease severity and progression. The molecular underpinnings of the Met-allele epistatic effect on the IL-1β-miR-142-3p axis and on its detrimental action on disease course need to be further investigated. However, this effect could be possibly attributed to the role of both BDNF and miRNA in synaptic transmission.

It is well known from the literature that *BDNF* Val66Met is involved in an altered intracellular trafficking and secretion of mature BDNF protein in synaptic cleft, impairing synaptic plasticity [19,30]. It cannot be excluded that the Met-allele also influences the ability of BDNF to limit the excitotoxic effects of neuroinflammation when secreted from immune cells in RRMS patients in response to relapses and during remitting phases [31].

A variety of cells expresses miR-142-3p, moving from peripheral monocytes, neutrophils and lymphocytes [32,33,34,35] to infiltrating immune cells, microglia and astroglia [5,8,10]. Increasing evidence in various physiological and pathological conditions suggests the presence of miR-142-3p in neurons [7,36,37,38], likely originating from extracellular vesicles produced by immune cells [35,39].

Many target mRNAs of miR-142-3p participate in immune system-related pathways, such as lymphocyte differentiation, proliferation, and activation [40,41,42,43]. miR-142-3p regulates the post-transcriptional expression of multiple genes involved in kinase signaling (JAK-STAT, MAPK, and PKA pathways) in both immune and nervous systems [10,43,44].

Among other pathways, the dopamine pathway is also affected by miR-142-3p through the direct inhibition of Dopamine Receptor D1 (*Drd1*) mRNA [45], with a possible implication for nitric oxide release from microglia and brain functioning [45,46].

In recent years, we demonstrated that miR-142-3p can downregulate the protein translation of the glial glutamate–aspartate transporter (GLAST), causing a dysregulation in glutamatergic transmission and consequent excitotoxic synaptic damage in IL-1β-dependent manner [5]. Part of the synaptic action of miR-142-3p might be also mediated by inhibiting microglial *BDNF*, which acts downstream in the miRNA signal pathway [10]. Although miR-142-3p does not directly bind *Bdnf* mRNA [15], it mediates *Bdnf* downregulation by repressing *Camk2a*, and, thus, the phosphorylation of Cyclic AMP-responsive element-binding protein (CREB), a crucial transcriptional activator of *Bdnf* expression [10]. It cannot be excluded that this pathway acts in other immune and non-immune cells than microglia, including neurons [7,36,37,38].

The interplay between miR-142-3p and BDNF, with the former acting upstream of the latter, suggests why the synaptotoxic effects of miR-142-3p are fully manifested only in Val/Val genetic context, namely when the BDNF protein can be properly produced and directed to synapses. Conversely, in the presence of Val66Met mutation, pro-BDNF fails to be matured and secreted adequately, thereby disguising the miR-142-3p synaptotoxic effects of miR-142-3p on MS course which are likely, at least in part, dependent on *BDNF* inhibition. Future studies are needed in this sense.

To the best of our knowledge, our study represents the first exploration in this field and aligns seamlessly with a clinical landscape increasingly oriented toward precision medicine. A primary limitation of our research is the relatively small sample size of enrolled pwRRMS (*n* = 69 114). A second limitation of our cohort is that the median age at diagnosis of pwRRMS was significantly higher in Met-allele carriers than in Val/Val homozygotes. There was a similar difference, but not significant, in the disease duration. The age of onset of the disease was similar in both groups. Recently, it has been reported improved resilience of neurological functioning to brain aging has been observed in healthy individuals that carrying the Met-allele [47]. This aspect, translated to MS, might explain a potential delay in disease onset due to compensation from clinical symptoms. Future studies are needed to confirm this hypothesis.

Nevertheless, together with further investigations, it may contribute to laying the groundwork for a tailored therapeutic approach based on specific prognostic and predictive biomarkers, including the CSF levels of miR-142-3p and the Val66Met genetic variant. Furthermore, considering the reported modulation of miR-142-3p expression by MS disease-modifying therapies [6,32,48] and its association with treatment response [6], our findings may provide valuable insights to inform the development of personalized treatment strategies for managing MS.

## 4. Materials and Methods

### 4.1. Patients with RRMS (pwRRMS)

A cohort of 114 pwRRMS was recruited at Neurology Unity of Neuromed Research Institute in Pozzilli, Italy, to participate in this retrospective observational study (Table 1). All the patients were diagnosed on the basis of clinical, MRI and laboratory evidence, according to 2017 revision of the McDonald criteria [1]. The Ethics Committee of Neuromed Research Institute approved the study (CE 26 October 2017; NCT03217396) according to the Declaration of Helsinki. All patients gave written informed consent to participate in the study. At the time of diagnosis, clinical evaluation and brain and spine Magnetic Resonance Imaging (MRI) were performed. Age, sex, EEDSS, the MSSS, the ARMSS, the PI, the presence of radiological disease activity, and disease duration, measured as the interval between disease onset and diagnosis were included in the evaluation. MRI scan (1.5T) included the following sequences: dual-echo proton density, fluid-attenuated inversion recovery (FLAIR), T1-weighted spin-echo (SE), T2-weighted fast SE, and contrast-enhanced T1-weighted SE before and after intravenous gadolinium (Gd) infusion (0.2 mL/kg). Presence of Gd-enhancing (Gd^+^) lesions defined radiological activity at the time of diagnosis.

### 4.2. CSF Collection and Analysis

CSF was collected at the time of diagnosis, during hospitalization, by lumbar puncture (LP) for medical purpose. No corticosteroids or disease modifying therapies were administered before LP. Cell-free CSF samples were stored at −80 °C and later analyzed [6]. Briefly, the CSF miR-142-3p levels were detected by quantitative real-time PCR (QIAGEN, Hilden, Germany) and normalized to miR-204-5p [6]. Samples were analyzed in duplicate. CSF concentrations of IL-1β were determined by using a Bio-Plex multiplex cytokine assay (Bio-Rad Laboratories, Hercules, CA, USA) and expressed in picograms/milliliter (pg/mL) according to the standard curve. Samples were analyzed in triplicate.

### 4.3. SNP Val66Met Analysis

All enrolled patients underwent genotyping for *BDNF* SNP Val66Met (rs6265). A blood sample (200 μL) was collected at the time of diagnosis. *BDNF* region containing Val66Met polymorphism was amplified by polymerase chain reaction with the TaqMan method performed using the ABI-Prism 7900HT Sequence Detection System (Applied Biosystems, Foster City, CA, USA; sense primer: 5′-AACCTT-GACCCTGCAGAATG-3′; antisense primer: 5′-ATGGGATTGCACTTGGTCTC-3′) [17]. Sequencing analysis was performed by 10 ng of PCR products, purified with Agenocourt AMPure PCR Purification kit (Agenocourt Bioscience Corporation, Beverly, MA, USA) in accordance with manufacturer’s instructions, using 0.5 pmoles of the sequence primer (5′-AAACATCCGAGGACAAGGTG-3′) and the ABI PRISM BigDye Terminator v3.1 Ready Reaction Cycle Sequencing Kit (Applied Biosystem, Foster City, CA, USA). The sequencing product was purified in using CleanSEQ dye terminal removal kit (Agencourt Bioscience Corporation, Great Boston Area, New England, MA, USA) and run on the Applied Biosystems 3730 DNA Analyzer Instrument (Applied Biosystem, Foster City, CA, USA).

The allele and genotype frequencies of our cohort were compared to the Caucasian population dataset in NHLBI’s TOPMed-BRAVO database.

The Polymorphism Phenotyping v2 (PolyPhen-2) tool was used to predict the possible impact of Val66Met substitution [49]. The HumVar approach was utilized for distinguishing drastic mutation effects, commonly observed in Mendelian disease. The HumDiv approach was applied to evaluate a milder contribution of the genetic variant to pathogenicity in a polygenic or multifactorial context. Variants with scores from 0.0 to 0.15 are predicted to be benign, while a score higher than 0.15 indicates a potentially damaging genetic variant, especially if it ranges from 0.85 to 1.0.

### 4.4. Statistical Analysis

Shapiro–Wilk test was used to evaluate the normality distribution of continuous variables. Data were shown as mean (standard deviation, SD) or median (interquartile range, IQR). Spearman’s nonparametric correlation was used to test possible associations between variables. Categorical variables were presented as absolute (n) and relative frequency (%). Difference in continuous variables between the *BDNF* SNP groups was evaluated using the nonparametric Mann–Whitney test. Box plots were employed to highlight statistically significant differences between groups. To distinguish the pwRRMS with low miR-142-3p (Low miR) and the pwRRMS with high miR-142-3p levels in the CSF (High miR), the cut-off value of 0.010 was used [6]. A *p* value < 0.05 was considered statistically significant.

All the comparisons were performed using Prism GraphPad 9.0 and IBM SPSS Statistics 17.0. for Windows/Mac (IBM Corp., Armonk, NY, USA).

## Figures and Tables

**Table 1:** Demographic and clinical characteristics of patients with relapsing-remitting multiple sclerosis (pwRRMS).

	*n*	114
Age at MS onset, years	Median (IQR)	30.8 (24.6–40.9)
Age at diagnosis, years	Median (IQR)	34.4 (27.4–45.3)
Sex, F	*n* (%)	78 (68.4)
Radiological activity, yes	*n*/tot (%)	45/109 (41.3) ^§^
OCB, yes	*n*/tot (%)	98/114 (86)
Disease duration, months	Median (IQR)	6.7 (1.1–36.7)
EDSS, value	Median (IQR)	1.75 (1–2.5)
MSSS, value	Median (IQR)	3.1 (1.4–5.9)
ARMSS, value	Median (IQR)	3.3 (1.8–5.9)
PI, value	Median (IQR)	0.2 (0.02–1.0)

Legend: F (female); OCB (oligoclonal bands); EDSS (Expanded Disability Status Scale); IQR (interquartile range). ^§^ missing data.

**Table 2:** Clinical and demographic characteristics of Val/Val pwRRMS.

RR-MS	N	Val/Val (69)	Low miR-142-3p Levels (36)	High miR-142-3p Levels (33)	*p*
Age at diagnosis, years	Median (IQR)	31.3 (25.1–41.7)	31.2 (26.5–44.8)	31.3 (24.0–41.7)	0.581
Sex, F	N (%)	49 (71)	27 (75.0)	22 (66.7)	0.310
Radiological activity, yes	N/tot (%)	27/65 (41.5) ^§^	12/33 (36.4) ^§^	15/32 (46.9) ^§^	0.272
OCB, yes	N/tot (%)	62/69 (89.9)	32/36 (88.9)	30/33 (90.9)	0.550
Disease duration, years	Median (IQR)	4.1 (1–35.7)	4.3 (1.0–45.8)	4 (1.0–34.1)	0.738
EDSS, value	Median (IQR)	1.5 (1–3)	1 (0–2)	2 (1–3)	0.013 *
MSSS, value	Median (IQR)	3.3 (1.0–5.9)	2.4 (0.7–4.9)	5.9 (1.7–7.4)	0.022 *
ARMSS, value	Median (IQR)	3.5 (1.7–6.3)	3.0 (1.2–4.7)	4.1 (2.5–7.3)	0.026 *
PI, value	Median (IQR)	0.2 (0.0–1)	0.1 (0.0–0.7)	0.4 (0.1–2.0)	0.011 *
miR-142-3p	Median (IQR)	0.009 (0.004–0.02)	0.004 (0.001–0.007)	0.02 (0.010–0.040)	0.001 *

Legend: pwRRMS (patients with relapsing-remitting multiple sclerosis); F (female); OCB (oligoclonal bands); EDSS (Expanded Disability Status Scale); MSSS (Multiple Sclerosis Severity Scale); ARMSS (Age-Related Multiple Sclerosis Severity Score); PI (progression index); IQR (interquartile range). ^§^ missing data; * denotes *p* < 0.05.

**Table 3:** Clinical and demographic characteristics of pwRRMS carrying Met-allele.

RR-MS	N	Met-Allele Carriers (45)	Low miR-142-3p Levels (24)	High miR-142-3p Levels (21)	*p*
Age at diagnosis, years	Median (IQR)	39.8 (31.5–47.2)	43.0 (34.1–47.9)	34.3 (30.4–43.7)	0.195
Sex, F	N (%)	29 (64.4)	15 (62.5)	14 (66.7)	0.509
Radiological activity, yes	N/tot (%)	18/44 (40.9) ^§^	7/23 (30.4) ^§^	11/21 (52.4)	0.121
OCB, yes	N/tot (%)	36/45 (80)	20/24 (83.3)	16/21 (76.2)	0.410
Disease duration, years	Median (IQR)	12.7 (2.4–36.9)	18.0 (2.9–37.0)	12.7 (2.3–30.9)	0.480
EDSS, value	Median (IQR)	2 (1–2.5)	2 (1–3)	2 (2–2.5)	0.716
MSSS, value	Median (IQR)	2.9 (2.0–5.9)	3.3 (1.8–5.9)	2.4 (2.2–5.9)	0.973
ARMSS, value	Median (IQR)	3.1 (1.9–5.0)	3.0 (1.7–5.2)	3.1 (2.2–5.0)	0.794
PI, value	Median (IQR)	0.1 (0.0–0.6)	0.1 (0.0–0.7)	0.2 (0.1–0.4)	0.640
miR-142-3p	Median (IQR)	0.01 (0.003–0.2)	0.003 (0.001–0.006)	0.02 (0.010–0.040)	<0.001 *

Legend: RRMS (relapsing-remitting multiple sclerosis); F (female); EDSS (Expanded Disability Status Scale); OCB (oligoclonal bands); IQR (interquartile range). ^§^ missing data; * denotes *p* < 0.05.

## Data Availability

The datasets presented in this study are available in an online repository at https://repository.neuromed.it/index.php/f/63510 (accessed on 24 March 2024).
